# Calcium signaling facilitates chilling- and GA- induced dormancy release in tree peony

**DOI:** 10.3389/fpls.2024.1362804

**Published:** 2024-03-19

**Authors:** Weiling Gai, Chunying Liu, Mengjie Yang, Feng Li, Hua Xin, Shupeng Gai

**Affiliations:** ^1^ College of Agriculture, Qingdao Agricultural University, Qingdao, China; ^2^ University Key Laboratory of Plant Biotechnology in Shandong Province, Qingdao, China; ^3^ College of Life Sciences, Qingdao Agricultural University, Qingdao, China; ^4^ College of Landscape Architecture and Forestry, Qingdao Agricultural University, Qingdao, China

**Keywords:** tree peony, bud dormancy, calcium, subcellular distribution, ultrastructure

## Abstract

Calcium plays a crucial role in plant growth and development, yet little is known about its function in endodormancy regulation. Tree peony (*Paeonia suffruticosa*), characterized by compound buds and large flowers, is well-known for its ornamental and medicinal value. To break bud dormancy release is a prerequisite of flowering and forcing culture, particularly during the Spring Festival. In this study, the Ca^2+^ chelator EGTA and Ca^2+^ channel blocker LaCl_3_ were applied, resulting in a significant delay in budburst during both chilling- and gibberellin (GA)- induced dormancy release in a dosage-dependent manner. As expected, the retardation of bud break was recovered by the supplementation of 30 mM CaCl_2_, indicating a facilitating role of calcium in dormancy release. Accordingly, several calcium-sensor-encoding genes including *Calmodulin* (*CaM*) and *Ca^2+^-dependent protein kinases* (*CDPK*s) were significantly up-regulated by prolonged chilling and exogenous GAs. Ultrastructure observations revealed a decline in starch grains and the reopening of transport corridors following prolonged chilling. Calcium deposits were abundant in the cell walls and intercellular spaces at the early dormant stage but were enriched in the cytosol and nucleus before dormancy release. Additionally, several genes associated with dormancy release, including *EBB1*, *EBB3*, *SVP*, *GA20ox*, *RGL1*, *BG6*, and *BG9*, were differentially expressed after calcium blocking and recovery treatments, indicating that calcium might partially modulate dormancy release through GA and ABA pathways. Our findings provide novel insights into the mechanism of dormancy release and offer potential benefits for improving and perfecting forcing culture technology in tree peonies.

## Introduction

Endodormancy is an important adaptive strategy for surviving cold winters and has a significant impact on the maintenance and production of the plants in temperate and arctic regions. Endodormancy is usually induced by short days (SD) and/or low temperatures, while it can be released by sufficient chilling accumulation and various chemicals, such as gibberellin (GA), hydrogen cyanamide (HC), mineral oil, potassium nitrate, and 5-azacytidine (5-azaC) (Sagredo et al., 2005; de Carvalho et al., 2014; Zhang et al., 2020b; 2021). Phytochromes (PHYA, PHYB1, and PHYB2) have been identified as early-acting components involved in SD-induced bud growth cessation in poplar (Ingvarsson et al., 2006; Kozarewa et al., 2010), and the CONSTANS/FLOWERING LOCUS T (CO/FT) regulatory module plays a vital role downstream (Böhlenius et al., 2006). The resumption of bud growth requires the activation of endogenous GA synthesis and signaling, alongside the suppression of ABA synthesis and signaling pathway (Rinne et al., 2011; Gai et al., 2013; Singh et al., 2018; Azeez et al., 2021). Recent studies have also addressed the alteration of subcellular Ca^2+^ localization and calcium signaling-related genes during the dormancy process in several tree species (Jian et al., 1997; 2003; 2004; Pang et al., 2007).

Calcium, in the form of Ca^2+^, is not only an essential nutrient for plant growth and development, but also a crucial second messenger involved in responding to environmental stresses and developmental cues. The vacuole, endoplasmic reticulum (ER), and apoplast serve as the main Ca^2+^ storage compartments. Recent studies have also detected Ca^2+^ in the nucleus, chloroplast, mitochondria, peroxisomes, and the endomembrane system, all of which play important roles in calcium signaling (Pirayesh et al., 2021).

In the signaling processes, a stimulus first induces transient or sustained increases in free cytosolic Ca^2+^ concentration ([Ca^2+^]), and the fluctuation in [Ca^2+^] is perceived by Ca^2+^ sensor proteins to trigger downstream responses. Finally, the excess free Ca^2+^ is then removed from the compartments where the Ca^2+^ transient appeared (Pirayesh et al., 2021). The oscillation of [Ca^2+^] is interactively controlled by a set of Ca^2+^ influx channels and efflux transporters located in the plasma membrane and the membranes of cellular organelles (McAinsh and Pittman, 2009; Demidchik et al., 2018). There are several types of calcium sensors in plants, such as calmodulin (CaM), calmodulin-like-proteins (CMLs), and Ca^2+^-dependent protein kinases (CDPKs), as well as calcineurin B-like proteins (CBLs) and their interacting kinases (CIPKs) (DeFalco et al., 2009; Mohanta et al., 2019). After perceiving the [Ca^2+^] oscillation, Ca^2+^ sensors function to regulate the associated physiological processes (DeFalco et al., 2009).

Early in 1993, it was reported that a low-temperature-induced Ca^2+^ influx is necessary for the expression of cold-acclimation-specific genes and the development of freezing tolerance in alfalfa (Monroy et al., 1993; Monroy and Dhindsa, 1995). Exogenous application of CaCl_2_ on dormant buds improves the quality of sweet cherry fruit at harvest (Michailidis et al., 2021). In *Populus*, leaf Ca^2+^ concentration significantly elevates prior to bud dormancy and leaf defoliation, and an increase of Ca^2+^ concentration in xylem sap is observed during the winter season (Furukawa et al., 2012). SD-induced alterations in subcellular Ca^2+^ localization and ultrastructural changes in plasmodesmata are closely associated with the development of dormancy in poplar buds (Jian et al., 1997; 2000). Pang et al. (2007) demonstrated that Ca^2+^ signaling is involved in HC-induced dormancy release in grape buds. Is calcium signaling also involved in chilling- and GAs-induced bud break in tree peony, a typical plant with compound buds and large flowers? Transcriptome analysis has shown that *CaM* and *CDPK* are differentially expressed during chilling accumulation (Gai et al., 2013), and the calcium signaling pathway is enriched in GAs-induced dormancy release (Zhang et al., 2021), indicating that calcium might be involved in dormancy regulation in tree peony. However, little is known about how calcium signaling affects bud dormancy release in tree peony.

Tree peony (*Paeonia suffruticosa* Andr.) is a woody shrub of the section *Moutan*, genus *Paeonia*, family Paeoniaceae, with high ornamental and medicinal value. It is also a newly cultivated oil plant. As a perennial deciduous plant, tree peony forms buds and undergoes endodormancy in late autumn to acclimate to cold and survive through winter. Breaking endodormancy is essential for budbreak, regrowth, and flowering under favorable conditions. Forcing culture constitutes a significant sector in the tree peony industry, and the successful release of endodormancy directly affects its ornamental and economic value. Agrotechnical measures, including sufficient chilling accumulation and chilling duration combined with gibberellin feeding, are widely applied to break dormancy in the forcing culture of peonies (Huang et al., 2008; Zhang et al., 2021). However, due to a poor understanding of the dormancy release mechanism, there are still many production problems such as flower abortion, branch shortening, and abnormal leaf and flower formation, which greatly reduce production value and hinder the development of the tree peony industry (Huang et al., 2008). Therefore, further research and improved strategies are necessary to increase production value and gain a deeper understanding of the dormancy release mechanism.

In this paper, we demonstrate that calcium acts in dormancy release processes using Ca^2+^ chelator and Ca^2+^ channel blockers. The distribution of calcium and the expressions of calcium sensor and dormancy release-associated genes were also tracked throughout the dormancy release processes. Our results will improve current understanding of the roles of Ca^2+^ and its regulation mechanism in the bud break process.

## Materials and methods

### Plant materials and growth conditions

Four-year-old tree peony plants (*P. suffruticosa* ‘Lu He Hong’) were obtained from the Tree Peony Research Institute of Qingdao Agricultural University, Qingdao, China, and planted in pots with a diameter of 38 cm and a height of 32 cm on Oct 15, 2019. The plants were moved to a 0–4°C dark refrigerating chamber on Nov 12 when the buds entered dormancy and underwent variable days of chilling (DC) (0, 7, 14, 21, and 28 DC). The chilled buds were collected for transmission electron microscopy (TEM) observation and gene expression analysis. The morphological characteristics of the chilled plants were evaluated to assess the dormancy status after being transferred to a greenhouse (18-22°C, 16-h-light/8-h-dark cycle) as described previously (Xin et al., 2019). In the experiment, 0-7 DC was a chilling perception period with less than 10% bud burst, 14-21 DC was a transition to dormancy release, 21 DC was completely dormancy release with all apical buds burst in greenhouse, and 28 DC was an ecodormancy state.

After this, 0.6 mM exogenous GA_3_ and GA_4_ were applied respectively, and the samples treated with sterile distilled water were used as a control group. Apical buds were harvested after 48 h for reverse transcription quantitative PCR (RT-qPCR) analysis. Three biological repeats were set for each treatment, and no less than nine plants were used per replicate.

### Calcium-blocking and recovery treatments during chilling- induced dormancy release

The calcium-blocking treatments were performed by spraying the calcium channel blocker lanthanum trichloride (LaCl_3_) or the calcium chelator ethylene glycol-bis (β-aminoethylether)-N, N, N´, N´-tetraacetic acid (EGTA) to buds of tree peony on Nov 11. Then the recovery treatments were executed by application of Ca^2+^ solution after the removal of LaCl_3_ or EGTA solution by ddH_2_O washing. Nine tree peony plants were included in each treatment and set up in triplicate.

For the blocking treatments, the buds of tree peony were sprayed with 10 mM and 30 mM EGTA or LaCl_3_, respectively, and then washed with double-distilled water (ddH_2_O) after 12 h. For the recovery treatments, the buds treated with LaCl_3_ or EGTA were sprayed with 10 mM or 30 mM CaCl_2_ solutions after being washed with ddH_2_O. After 12 h, all the treated plants were moved to a refrigeration room (0-4°C) for 21 d, and then transferred to the greenhouse (18-22°C, 16 h light and 8 h darkness). The percentages of bud break were monitored daily to assess the effect of the treatments on dormancy release. Tree peonies sprayed with H_2_O alone served as the control.

### Calcium-blocking and recovery treatments during GA- induced dormancy release

In the blocking treatments, the tree peony buds were sprayed with 30 mM LaCl_3_ or EGTA and washed with ddH_2_O as described above, and then 0.6 mM GA_3_ was applied. For the recovery treatments, 10 mM or 30 mM CaCl_2_ was sprayed after treated with LaCl_3_ or EGTA, along with GA_3_. Subsequently, all the GA-related plants were immediately moved to the greenhouse, and the bud break rate was monitored. The plants treated with 0.6 mM GA_3_ alone served as the control. Three replicates were performed as described above.

The differences of morphological data were analyzed using Duncan’s multiple range tests at a significance level of 0.05 using SPSS 13.0 for Windows (SPSS, USA).

### Cytochemical localization of calcium

Buds were collected at 11:00 a.m. from plants treated with different chilling days (0, 7, 14, 21, and 28 DC). The leaf primordia from the same location in different samples were cut into a cube with a volume of approximately 1 mm^3^ and prepared for calcium localization, as described by Jian et al. (1997) with minor modifications. The samples were immersed in a fixative solution containing potassium pyroantimonate, glutaraldehyde, and paraformaldehyde in potassium phosphate buffer, post-fixed by osmium tetroxide, dehydrated in a graded series of acetone, and embedded in Embed*_*812 (EMS, New Jersey, USA). The embedded samples were then sectioned with an Ultra microtome, and the sections were 60-70 nm thick.

After uranyl acetate double staining, transmission electron microscopy images about cell structure were acquired using a HITACHI 7700 microscope (HT7700). In order to verify the location of calcium, an additional chelation of calcium ion with EGTA treatment was performed (Wick and Hepler, 1982). The grids were immersed in a 200 mg/L EGTA solution, incubated at 60°C for 1 h, and subsequently rinsed briefly in distilled water. After EGTA treatment, the samples were restained with uranyl acetate and reexamined using TEM.

### RNA isolation and reverse transcription quantitative PCR polymerase chain reaction

Total RNA was extracted using the TRIZOL reagent (Qiagen) following the manufacturer’s instructions, and RNA integrity was checked by an Agilent Bioanalyzer 2100 (Agilent technologies, US). Chromosomal DNA was removed with RNase-free DNase (Fermentas, USA). First-strand cDNA was synthesized from 2 μg of total RNA using the PrimerScript™ RT reagent Kit (Takara, Dalian, China) according to the manual.

Differential expressed calcium-sensor-encoding genes were screened from the tree peony transcriptional database of chilling duration (accession to the GEO data: GSE4004) and GA- induced dormancy release (accession to the SRA data: PRJNA720276) (Gai et al., 2013; Zhang et al., 2021).

The expression patterns of target genes during dormancy release and chemical substance treatment were analyzed using RT-qPCR. The PCR reactions were performed in 25 μL volume, containing 12.5 μL of 2× SYBR Green Master mix (Takara), 0.75 μL of each primer, 9 μL of ddH_2_O, and 2 μL of 10× diluted cDNA template. The PCR reactions were conducted in a Roche LightCycler^®^ 480 (Roche, Germany) using the following program: 95°C for 2 min, followed by 45 cycles of 95°C for 5 s, 57°C for 30 s, and 72°C for 30 s. *Actin* was used as a reference gene to normalize the RT-qPCR results. The reactions were performed in triplicate. The relative expression of each gene was quantified using the 2^-ΔΔCt^ method as described by Livak and Schmittgen (2001). Significance was tested using SPSS 13.0 for Windows (SPSS, USA). The primers used for qPCR are listed in **Supplementary Table S1**.

## Results

### Ca^2+^ is involved in endodormancy release induced by chilling

Most of the control buds chilled for 21 and 28 days burst after two weeks in the greenhouse and eventually blossomed, while only some of the buds chilled for 0-14 days burst in two weeks. The state after 21 d indicated a dormancy release stage, and that of 28 d indicated ecodormancy status.

**Figure 1:**
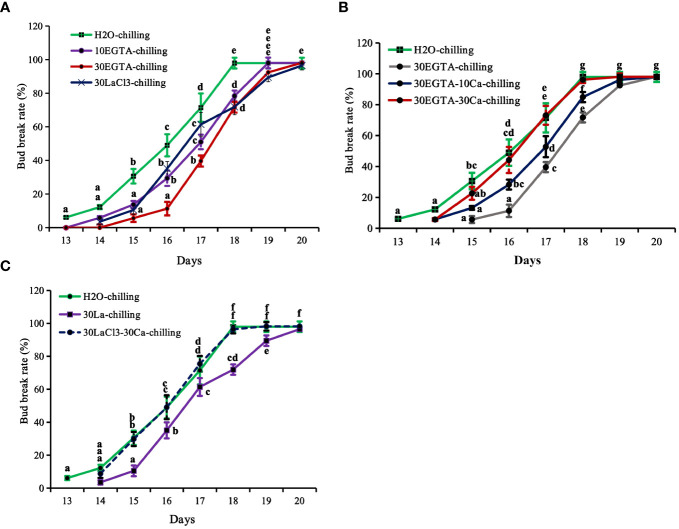
The breaking percentage of tree peony ‘Lu He Hong’ buds in the greenhouse after being treated with controlled chilling for 21 days in a 0-4°C refrigerating chamber. The buds sprayed with ddH_2_O only served as the control. All treated tree peonies were placed in a greenhouse (18-22°C,16 h/8 h light/dark). Bud breaking percentages were calculated at 11:00 every day during the forcing period. The values were the averages of three replicates, with nine plants per replicate, and the bars represented the standard deviations. **(A)** The breaking percentages for the calcium-blocking treatments with the calcium chelator EGTA and the calcium channel blocker LaCl_3_, respectively. **(B)** The breaking percentages for the recovery treatments with different concentrations of Ca^2+^ solution after removal of EGTA solution. **(C)** The breaking percentages for the recovery treatments with 30 mM Ca^2+^ solution after removal of LaCl_3_ solution. Days in the horizontal axis represented time after transfer to the greenhouse. Letters indicated the significant differences (one-way ANOVA, Duncan’s multiple range test).

The calcium blocking treatments EGTA and LaCl_3_ delayed first bud burst and reduced bud break rates, with the most inhibitory effect obtained with 30 mM EGTA treatment application before 18 d. On average, the buds treated with 30 mM EGTA required an additional day to reach maximum burst. The control buds were on full flushing 18 d after being transferred to the greenhouse, while the bud break rates of 10 mM EGTA-, 30 mM EGTA-, and 30 mM LaCl_3_-treatments were 78.43%, 71.70%, and 71.93%, respectively (**Figure 1A**). Despite the delays, the buds of all treatments eventually burst.

Recovery treatments with both 10 mM and 30 mM Ca^2+^ solution promoted bud burst before 19 d, reducing 1.2 d compared to the buds in the 30 mM EGTA treatment (**Figure 1B**). Comparatively, the recovery effect of 30 mM Ca^2+^ was superior to that of the 10 mM Ca^2+^ solution. At 18 d, the bud break rate of the 10 mM Ca^2+^-treated group was 84.91%, while that of the 30 mM Ca^2+^- group was almost full flushing, nearly identical to that of the H_2_O control from 14 d to 20 d after being moved to the greenhouse, with the only exception being a 1.1 d delay at beginning of bud burst compared to the control (**Figure 1C**).

### Ca^2+^ plays an important role during the dormancy release induced by GA_3_

**Figure 2:**
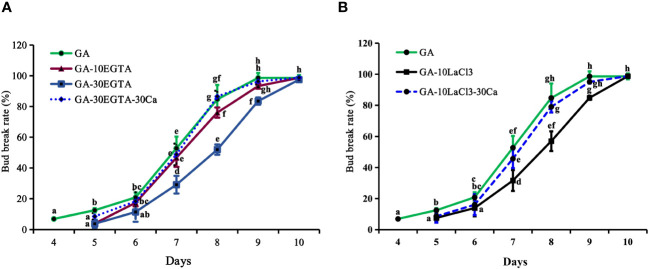
The breaking percentages of tree peony ‘Lu He Hong’ buds in the greenhouse after being treated with GA_3_. The buds sprayed with 0.6 mM GA_3_ only were considered as the control. All treated tree peonies were placed in the greenhouse (18-22°C,16 h/8 h light/dark). Bud breaking percentages was calculated at 11:00 a.m. daily during the forcing period. The values were the averages of three replicates, with nine plants per replicate, and the bars represented the standard deviation. **(A, B)** displayed the breaking percentages for calcium-blocking treatments with the calcium chelator EGTA and the calcium channel blocker LaCl_3_, and the recovery treatments by spraying 30 mM Ca^2+^ solution after removal of EGTA or LaCl_3_ solution, respectively. Days in the horizontal axis represented time after transfer to the greenhouse. Letters indicated the significant differences (one-way ANOVA, Duncan’s multiple range test).

Bud break first occurred after 4 d of GA_3_ feeding, which was about 9 d earlier than that with chilling treatment alone (**Figures 1**, **2**). The buds treated with EGTA and LaCl_3_ showed delayed bud burst and declined breaking percentages for up to 9 days in the greenhouse, and the buds of all treatments eventually burst. After 9 days in the greenhouse, the mock was full flushing and the breaking rate of 10 mM EGTA was 93.01% with no significant difference to that of the mock, while that of 30 mM EGTA dramatically decreased with a percentage of 83.54%. The results indicated that application of EGTA delayed bud break in a concentration-dependent manner (**Figure 2A**). Unlike the chilling groups, the inhibitory effect of 10 mM LaCl_3_ was almost the same as that of the 30 mM LaCl_3_ and the breaking percentages of 10 mM LaCl_3_ and 30 mM LaCl_3_ treatments were lower than that of the control (**Figure 2B**).

The inhibitory effect of calcium blockage was eliminated after 5 to 10 days in the greenhouse when 30 mM Ca^2+^ was supplied following EGTA or LaCl_3_ treatment, as no significant differences were observed between the two groups during this period. However, the buds in the calcium recovery group burst one day later than those in the mock.

### Cytochemical localization of calcium

**Figure 3:**
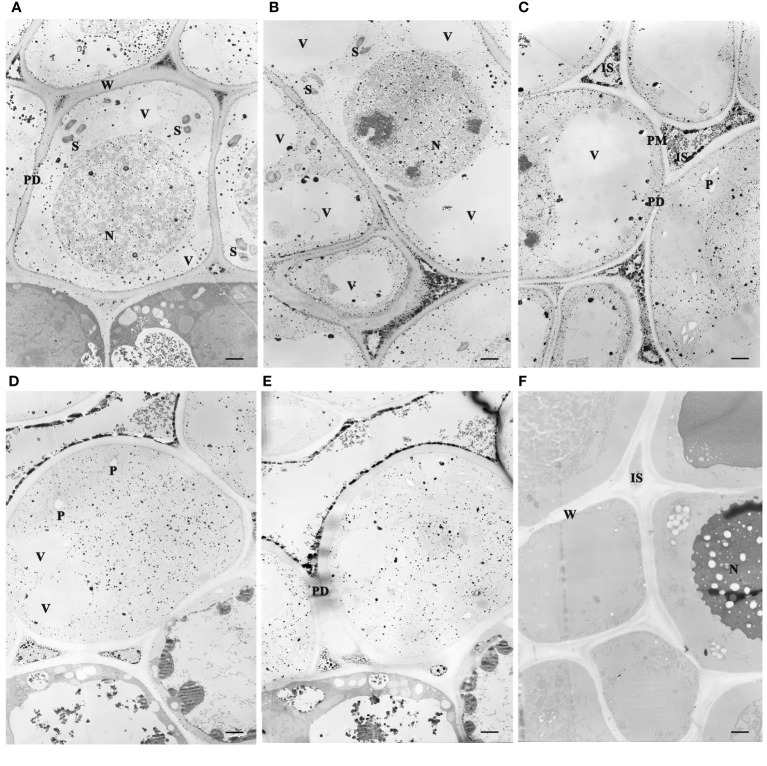
Subcellular localization of Ca^2+^ antimonate deposited in the juvenile leaves of the apical bud revealed by TEM (3,000×). The tissues were fixed using potassium pyroantimonate-containing solution and stained with uranyl acetate double staining after ultramicrotomy. **(A–E)** represented TEM observations after chilling duration for different days: 0, 7, 14, 21, and 28 DC. **(F)** Tissue sections treated with EGTA. S, Starch. N, Nucleus. P, Plastid. IS, intercellular space. W, cell wall. ER, Endoplasmic reticulum. PD, plasmodesmata. PM, plasma membrane. V, vacuoles. Bar=1 µm.

Dormancy release was accompanied by variations of ultrastructure and calcium distribution, gradual disappearance of starch grain, and the reopening of the transport channel. Mesophyll parenchyma cells were relatively small in size and had comparatively large nuclei centrally located in the cells, as well as several small vacuoles at the early dormant period (**Figures 3A, B**). Mitochondria, plastids, endoplasmic reticulum, and ribosomes presented in the cytoplasm, and the plastids were not well developed. Upon dormancy release (21 and 28 DC), the cells enlarged obviously compared to those chilled for 0 d. During the early dormant period, abundant starch grains accumulated in the apical bud cells and gradually diminished with prolonged chilling. In the deep dormant state, the plasmodesmata in the cell walls were blurred, and the entrances of the plasmodesmata appeared to fuse with each other, forming a continuous membrane (**Figures 3A–C**). At 21 and 28 DC, the plasmodesmata were distinct in the walls of adjacent cells (**Figures 3D, E**).

Electron microscopic observations revealed that the sample sections immobilized by the fixative solution containing potassium antimonite presented electron-dense deposits in the cells (**Figures 3A–E**). The calcium antimonite precipitates were clearly localized in the intercellular spaces, cell walls, and cytosols. After EGTA chelation, most granules disappeared, accompanied by legible holes presented in the EGTA-treated section (**Figure 3F**), where the Ca^2+^ antimonate precipitate might be before EGTA chelation. This suggests that the electron-dense deposits were calcium precipitates and were indicative of subcellular Ca^2+^ localization in tree peony buds.

Prolonged chilling triggered a Ca^2+^ influx to the cytosol and nucleus from the cell wall and intercellular space. Ca^2+^ deposits were prominently observed in the intercellular spaces throughout the whole chilling duration period, but were notably scarce in the vacuoles, possibly owing to the inadequate development of vacuoles for storing Ca^2+^. At 0 and 7 DC, the most intense Ca^2+^ signals were localized in the intercellular spaces and cell walls, and some granules existed in the nucleus, cytosol, plastid, and other organelles (**Figures 3A, B**). Along with chilling accumulation, the Ca^2+^ deposits in intercellular space and cell wall gradually decreased, while there was an increase in the nucleus, cytosol, and endoplasmic reticulum. At 14 DC, most of the calcium precipitates in the cell wall had disappeared, and dense Ca^2+^ granules were observed congregating along the plasmalemma (**Figure 3C**), suggesting a Ca^2+^ influx from intercellular to intracellular regions. At 21 and 28 DC, calcium signals in the cell wall almost completely vanished, and only a few residual signals remained in the intercellular spaces (**Figures 3D, E**).

### Expression patterns of calcium sensor encoding genes

**Figure 4:**
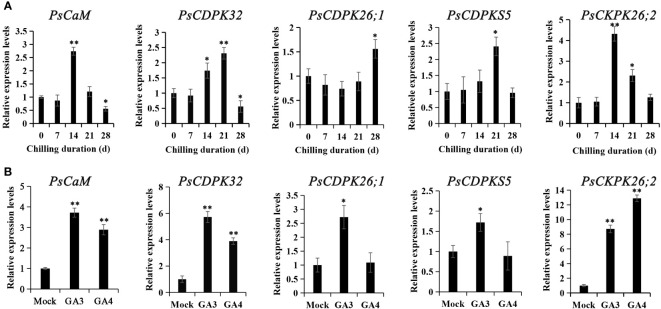
The expression patterns of genes encoding calcium sensors during chilling-**(A)** and GA-**(B)** induced dormancy processes. Chilled buds were sampled immediately after 0, 7, 14, 21, and 28 DC. GA feeding buds were harvested 48 h after exposure, and RT-qPCR was used to evaluate their expression patterns. Data were represented as the mean ± standard deviation (SD) of three biological replicates. *ANOVA; Tukey test, P < 0.05. **P < 0.01.

Putative calcium sensor encoding genes involved in chilling- and GA- induced dormancy release were identified from the previous transcriptional database. One CaM homolog (Transcript_34059) and four CDPK homologs (transcript_12835, 13256, 16356, and 3925) were significantly differentially expressed both during chilling duration and GA-exposure according to the RNA-seq data (Gai et al., 2013; Zhang et al., 2021). These were chosen as candidates for RT-qPCR analysis. Transcript_34059 (*PsCaM*), transcript_12835 (*PsCDPK32*), and transcript_3925 (*PsCDPK26;2*) were induced at 14 d of chilling treatment, while transcript_16356 (*PsCDPKS5*) and transcript_12835 (*PsCDPK32*) peaked at 21 d of chilling, and transcript_13256 (*PsCDPK26;1*) was not up-regulated until 28 DC. The calcium sensor encoding genes significantly declined after reaching a peak, except for transcript_13256 (**Figure 4**). The changes in expression patterns suggested that transcript_34059, 12835, 3925, and 16356 might participate in chilling-induced dormancy release, while transcript_13256 was not associated with dormancy release.

Subsequently, the expression patterns of the calcium sensor encoding genes were detected after GA_3_ and GA_4_ feedings. All the candidate calcium sensor encoding genes were significantly upregulated by GA_3_ treatment, whereas GA_4_ treatment only enhanced the expressions of transcript_34059, 12835, and 3925. Notably, transcript_3925 was the most dramatically activated gene by GA feedings. In most cases, GA_3_ treatment led to higher levels of gene expression than GA_4_, with the exception of transcript_3925 (**Figure 4B**). The changes in expression patterns after GA_3_ and GA_4_ feeding suggested that transcript_34059 (PsCaM), 12835 (PsCDPK32), 3925 (PsCDPK26;2), and 16356 (PsCDPKS5) were involved in chilling- and GA-induced dormancy release in tree peony.

### Calcium oscillation influenced the expression of dormancy release-associated genes

**Figure 5:**
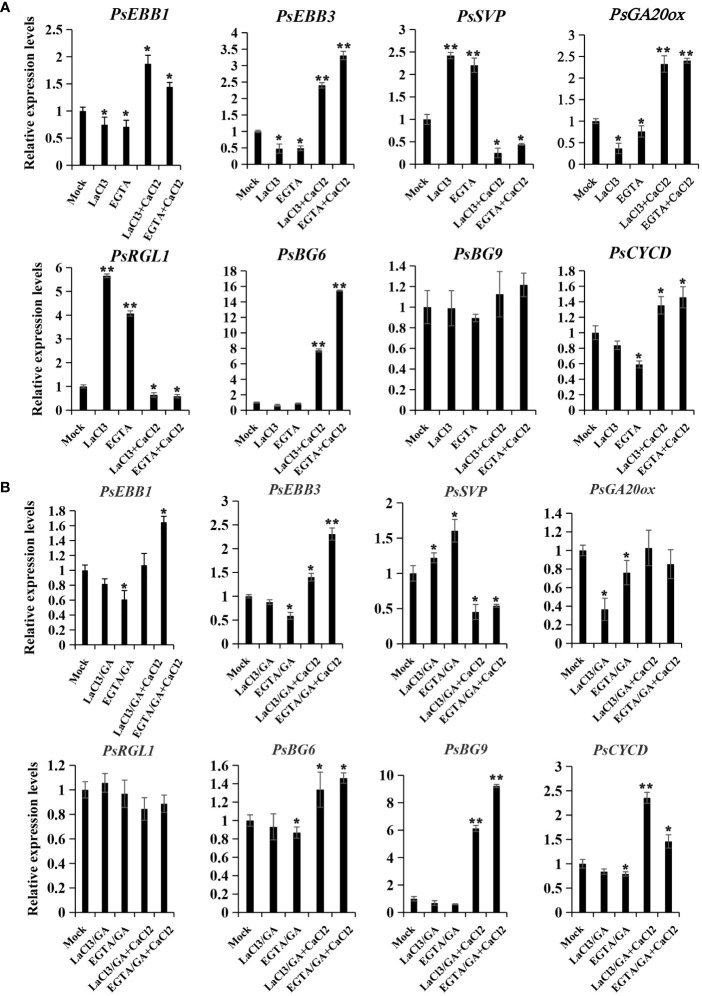
The expression patterns of dormancy release-associated genes after calcium treatment in chilling-**(A)** and GA-**(B)** groups. Calcium blocking and recovery treatment coupled with chilling or GA exposure were performed, and the buds were sampled after treatment with chemicals for 72 h, and qPCR was used to evaluate their expression patterns. Data were represented as the mean ± standard deviation (SD) of three biological replicates (ANOVA; Tukey test, *P < 0.05, **P< 0.01).

To elucidate the potential role of calcium in modulating dormancy release, we examined the expression patterns of genes associated with dormancy release following calcium treatments, including PsEBB1 (Zhang et al., 2023b), PsEBB3, PsSVP, PsRGL (Gao et al., 2023), PsGA20ox, PsCYCD (Gai et al., 2013), PsBG6, and PsBG9 (Gao et al., 2021). In the chilling group, PsEBB1, PsEBB3, PsGA20ox, PsCYCD, and PsBG6 displayed similar expression patterns, being inhibited in the calcium blocking subgroup and reawakened in the calcium recovery subgroups. Conversely, the expressions of PsSVP and PsRGL, negative regulators of dormancy release, increased through calcium blocking but decreased with replenishment of CaCl_2_ (**Figure 5**). PsBG9 exhibited minimal response to calcium fluctuation in the chilling group.

In the GA treatment group, PsEBB1, PsEBB3, and PsCYCD were also inhibited by calcium blocking and recovered by supplement of CaCl_2_, while PsSVP displayed the opposite trend. PsGA20ox was suppressed by calcium blocking but could not be recovered by calcium supplement, whereas PsRGL was relatively stable compared to the mock. Different to the chilling group, PsBG9 was down-regulated by calcium blocking, but was sharply and significantly up-regulated by CaCl_2_ recovery, while PsBG6 was unaffected by LaCl_3_ and slightly increased with CaCl_2_ treatment (**Figure 5B**). The differing responses of PsBGs to calcium treatment between the two groups might be associated with their sensitivity to chilling and GAs.

## Discussion

An influx of Ca^2+^ from the intercellular space and the cell wall contributes to chilling-induced dormancy release in tree peony. Our study provides some critical insight into the pivotal role of Ca^2+^ in dormancy release, partially through its interaction with GA and ABA pathways.

### Prolonged chilling triggers ultrastructure changes and Ca^2+^ influx in peony bud

Both dormancy induction and release undergo obvious ultrastructural changes to accommodate changes in growth signals. During the development of dormancy, several ultrastructural changes take place in the Shoot Apical Meristem (SAM) cells of the bud. In Populus, the number of starch granules increases, and the plasmodesmata are gradually blocked along with SD exposure (Jian et al., 1997; Rinne et al., 2001; Ruonala et al., 2008). However, there is limited documentation of the ultrastructural variations in buds related to dormancy release. After exposure to prolonged chilling, the number of starch grains gradually reduced and became almost undetectable by the end of dormancy in tree peony buds. The result coincided with the activation of starch hydrolysis and carbohydrate metabolism in the dormancy release process of tree peony (Zhang et al., 2018; 2020a), which will provide sufficient substance and energy supplements for bud dormancy release and subsequential bud burst. Simultaneously, the constriction and blockage of plasmodesmata were relieved at the dormancy release stage (**Figures 3D, E**), indicating a reopening of the transport corridor to transmit growth signal and nutrient substance. Recently, we identified that PsBG6 and PsBG9 are induced by chilling and exogenous GAs to break down callose deposited on the PD, respectively (Gao et al., 2021), which might contribute to the process. By comparison, dormancy release and dormancy induction seemed to undergo opposite ultrastructural changes when the number of starch granules and the status of plasmodesmata were concerned.

The variations of subcellular Ca^2+^ distribution indicated that prolonged chilling triggered a Ca^2+^ influx to the cytosol and nucleus, which contributed to dormancy release in tree peony. The relationship between cellular Ca^2+^ distribution and dormancy induction has been extensively reported (DeHayes et al., 1997; Jian et al., 1997; 2004), while little is known about the dormancy release process. Our results presented the dynamic changes of calcium distribution for the first time during the chilling-induced dormancy release process. Initially, numerous Ca^2+^ deposits were found in the intercellular spaces and cell wall of the tree peony bud, which is consistent with findings in poplar SAM cells after 77 d SD exposure (Jian et al., 1997). Additionally, the genes related to calcium sensors, CaM and CDPK, were up-regulated during this period. The results suggested an increased influx of Ca^2+^ translocated from the intercellular space into the inner side of the cell, resulting in an increase of [Ca^2+^]_cyt_. Under long day conditions, cellular calcium is mainly localized in vacuoles, intercellular spaces, and plastids in poplar. With prolonged SD exposure, there is an increased presence of Ca^2+^ deposits in the cytosol and nuclei, while numerous Ca^2+^ precipitates reappear in the cell walls and intercellular spaces at a deep dormant status (Jian et al., 1997). Together, the process of dormancy release exhibited a similar variation in calcium redistribution as observed during dormancy induction. Lautner and Fromm (2010) documented a marked increase of [Ca^2+^] in meristem after dormancy break in trees, but we speculated that it was the increase of [Ca^2+^]_cyt_, rather than the tissue calcium content, associated with dormancy release in deciduous plants.

Additionally, the influx of Ca^2+^ suggests the need for investigation and function analysis of the candidate Ca^2+^ influx channels and efflux transporters during chilling duration, which will be benefit the understanding of the activation and modulation of calcium signals during the dormancy release process.

### Calcium is positively involved in chilling- and GA-induced dormancy release

In tree peony, LaCl_3_ and EGTA feedings dramatically delayed bud break in both chilling- and GA-induced dormancy release, while exogenous application of CaCl_2_ mitigated the retardation, which was similar to findings in grape (Pang et al., 2007). Additionally, the genes encoding calcium sensors, such as CaM (transcript_34059) and CDPK (transcript_34059, 39255 and 16356), were significantly induced prior to bud dormancy release, and they were further up-regulated by exogenous GA feeding. The up-regulation of calcium-sensor-associated genes confirmed that an increase of [Ca^2+^]_cyt_ and an activation of calcium signaling were triggered by prolonged chilling and exogenous GAs.

Recent research has indicated the involvement of calcium in bud dormancy regulation. Firstly, calcium contents undergo alterations during the dormancy induction and release processes (Lautner and Fromm, 2010; Furukawa et al., 2012). Secondly, the calcium signaling pathway has been identified as enriched in the differentially expressed genes between dormancy and dormancy release phases (Anderson et al., 2005; Zhang et al., 2021). Most importantly, exogenous application of calcium and calcium blocking reagents influence dormancy status (Weis et al., 1999; Pang et al., 2007). Collectively, these findings lead us to speculate that activation of calcium signaling promoted bud dormancy release in tree peony.

### The potential mechanism of calcium in bud dormancy release

Calcium blocking delayed bud break, while calcium recovery treatments promoted bud break in chilling- and GA-induced dormancy release in tree peony. The results help to elucidate the mechanism by which calcium regulates dormancy release through the GA and ABA pathway. GA20OX, a key GA biosynthesis gene, contributes to bioactive GA synthesis and dormancy release (Gai et al., 2013; Zhang et al., 2020a), and RGL1 is a negative regulator of the GA pathway, suppressing dormancy release in tree peony (Gao et al., 2023). EBB1, SVP-like (SVL), EBB3, and CYCD3 represent a network of ABA pathway to modulate dormancy release in hybrid poplars (Singh et al., 2018; Azeez et al., 2021). As shown, treatments with calcium blockers depressed the expressions of PsEBB1, PsEBB3, PsGA20OX, and PsCYCD, while promoting PsSVP and PsRGL1 transcripts during dormancy release induced by chilling and GA. Conversely, replenishing calcium either increased or decreased their expressions. The results indicated that calcium blocking to reduce calcium content might suppress bioactive GA synthesis, and promote ABA biosynthesis and signaling, while calcium recovery to free calcium compensated for the imperfection.

Calcium signaling modulating GA and ABA pathways has been documented in several plants and physiological processes (Kim et al., 2010; Ho et al., 2013; Zhang et al., 2023a). In soybean, low concentrations of CaCl_2_ inhibit GA biosynthesis and impede radical protrusion, whereas high concentrations of CaCl_2_ suppress ABA biosynthesis (Wang et al., 2021). The external application of calcium nitrate induces gibberellin biosynthesis and signal transduction, thereby promoting stem elongation of Dendrobium officinale (Du et al., 2023). Taken together, we assumed that calcium might facilitate dormancy release by modulating the GA and ABA signaling pathways, namely activating GA biosynthesis and signaling, and depressing ABA biosynthesis and signaling.

Additionally, PsBG6 and PsBG9 were also significantly up-regulated by calcium recovery in chilling- or GA- induced dormancy process. The results hinted that fluctuation in free [Ca^2+^] modulates the reopening of transport channels in tree peony. Further study to investigate how calcium triggers GA biosynthesis and the expression of PsBG6 and PsBG9 will be helpful in understanding the mechanism of calcium facilitating dormancy release in tree peony.

The mechanism of calcium modulating dormancy release has been poorly described up to now. We screened several PsCDPKs involved in the regulation of dormancy release. In wheat, TaCDPK30 interacts with TabZIP60 to regulate salt tolerance via modulating ABA synthesis (Zhang et al., 2023a). We speculated that PsCDPKs might function through the phosphorylation of downstream proteins to convey the calcium signal. Further investigations on PsCDPK-interacting proteins will be helpful in understanding the transduction of calcium signals during chilling- and GA-induced dormancy in deciduous plants.

In summary, we confirmed the positive role of calcium in bud dormancy release in tree peony. Prolonged chilling resulted in a calcium influx from the intercellular space and cell wall to the cytosol and nucleus, which was perceived by calcium sensors such as CaM and CDPK, leading to the activation of calcium signaling. Subsequently, biosynthesis of bioactive GAs was stimulated, while the ABA pathway was inhibited. Ultimately, dormancy was broken along with the activation of the cell cycle. These results presented a potential mechanism through which calcium facilitates dormancy release, offering beneficial insights to tree peony production.

## Data availability statement

The original contributions presented in the study are included in the article/**Supplementary Material**. Further inquiries can be directed to the corresponding authors.

## Author contributions

WG: Writing – original draft, Validation, Methodology, Investigation. CL: Writing – original draft, Investigation, Data curation. MY: Writing – original draft, Methodology, Investigation, Formal analysis, Data curation. FL: Writing – original draft, Methodology, Investigation, Formal analysis, Data curation. HX: Writing – review & editing, Validation, Methodology, Conceptualization. SG: Writing – review & editing, Supervision, Project administration, Conceptualization.
