# Supplementary material for: Calcium signaling facilitates chilling- and GA- induced dormancy release in tree peony
Source: Front Plant Sci. 2024 Mar 19;15:1362804. doi: 10.3389/fpls.2024.1362804 (PMC10985203; doi:10.3389/fpls.2024.1362804)
Supplement: Supplementary file 1 [file Table_1.docx]

**Supplementary material**

Table S1 The primer information used in this study

| Name | Primer F (5'-3') | Primer R (5'-3') |
| --- | --- | --- |
| PsCYCD | GAGGCCGTGGATTGGATTCT | AAAAGGGGCACTTGGGTCTC |
| PsEBB1 | AATAGCCCGCGAAGTCCAAA | GGGATCTGATGAACCAGCCC |
| PsRGL1 | ACCACGCCAAGATTTAGATG | GTTGACTGAACTCGGTGAGG |
| PsEBB3 | GGTGAGATTACTCCGCCACC | CGACCCTGAATCTGAGACCG |
| PsBG6 | CCTACTACCCGGCCACAAAG | CTACTGAAAGCACCCGCAGA |
| PsBG9 | TCATCTTCGCTCCGATGCTC | TCGGCCACAACTATGTCCAC |
| PsGA20ox | AGTGTTTGTAGACGATGAGTG | CCTGGGATTGTTATTAGTGTCC |
| PsSVP | GCACATGGTTTGCGAGGAAG | CCCGAATATGGCAGTCCCAA |
| trans_12835 | GGTGCTTGCTTGCAAATCCA | TCGGTGTAATGTCCCCTTGC |
| trans_13256 | GGCCTCAACTCTCAGCAACT | AGTTCCAAACTGTCCCTGGC |
| trans_16356 | CTGAGCTCATGGAGTCCGAG | GGTGGACCTCACTTAACCCG |
| trans_34059 | TGAGATCGTTGGGGCAGAAC | CATCACGTGACGCAATTCGG |
| trans_3925 | TGCGCCAGGTTCTCATTAGG | GCTACAAAGGGTTTGGCGTG |
| PsActin | GAGAGATTCCGTTGCCCTGA | CTCAGGAGGAGCAACCACC |
